# Distinctions in photochemistry between *Chlorella ohadii* and *Chlorella sorokiniana* are dependent on growth light intensity

**DOI:** 10.3389/fpls.2026.1858699

**Published:** 2026-07-29

**Authors:** Grant Steiner, Devinjeet Saini, Colin Gates

**Affiliations:** 1 Department of Chemistry and Biochemistry, Loyola University Chicago, Chicago, IL, United States; 2 Department of Bioinformatics, Loyola University Chicago, Chicago, IL, United States

**Keywords:** *Chlorella ohadii*, *Chlorella sorokiniana*, light intensity, photochemistry, photoprotection, quantum yield

## Abstract

*Chlorella ohadii*, currently the fastest-growing eukaryotic phototroph on record, is very closely related to the slower-growing *Chlorella sorokiniana*. *C. ohadii* also tolerates extreme light conditions in its normal growth and has been shown to perform better photoprotection comparatively. *C. ohadii* has previously been shown to tolerate this extreme light in part by performing cyclic electron flow around photosystem II. We have induced *C. sorokiniana* to grow rapidly under elevated CO_2_ and extreme light, reaching a doubling time of just under 1.7 hours at peak, and investigated how its photophysiological adaptations compared to the native behavior of *C. ohadii*. When grown in low light conditions, both strains have similar efficiency of their water oxidizing complex operation, but under extreme light conditions, we do not observe efficient oscillation of the water oxidizing complex in *C. sorokiniana*, indicating considerable recombination until exogenous electron acceptor is added. Similarly, there is a relatively higher rate of oxidation of the Q_B_ site, which can be explained by this recombination. However, the rate of photosystem II turnover is not distinguishable between the two species under these conditions, and total antenna expression is similar. Cytochrome *b_6_f* and plastocyanin stay significantly more reduced in extreme light grown *C. sorokiniana*, but PSI does not, implying elevated PSI-CEF as a more significant component of photoprotection in this species. A weaker initial electrochromic shift is observed in extreme light grown *C. sorokiniana*, but comparatively limited gradient breakdown precludes this being due to elevated ATP synthase activity. Overall, these discrepancies suggest the structural arrangement of the photosynthetic apparatus, specifically regarding photosystem II, is responsible for the improved efficiency and photoprotection of *C. ohadii* compared to its near relative.

## 1 Introduction

Green microalgae have provided excellent sources of biotechnical research models over the last several decades, for instance, offering remarkable insights into microbial symbiotic relationships and new means of resource sustainability (Spolaore et al., 2006; Oncel, 2013; Thoré et al., 2023). Microalgae and other unicellular phototrophs have also long been utilized for enhancing the nutritional value of human diets, due to their high protein, lipid, and carbohydrate contents (Spolaore et al., 2006; Chen et al., 2022). Various species and strains of phototrophs in these groups also accumulate carotenoids, antioxidants, and vitamins comparable to land crops (Milledge, 2011; Borowitzka, 2013; Wang et al., 2023). One frequently investigated genus in this ensemble is *Chlorella*, which was the organismal model behind the Nobel Prize winning research illustrating the assimilation of carbon dioxide that led to the elucidation of the Calvin cycle (Bassham et al., 1954). Because the original classifications of what organisms are included in the genus *Chlorella* were based on generalized morphological observations (green, coccoid cells with a diameter in the range of a few microns and a wide variety of pyrenoids and chloroplast shapes), the genus became somewhat of a “wastebasket taxon”, with hundreds of species that are now split between multiple genera, and even families (Araki and Takeda, 1992; Kessler and Huss, 1992; Baudelet et al., 2017). Via advancement of methods utilized in molecular phylogenetics over the last few decades, several species have been moved to or from the *Chlorella* clade quite frequently. As a result, it is difficult to presume the ubiquity of phenotypes presented in model organisms within this clade.

One example of a unique species within the *Chlorella* genus is the extremophile, *Chlorella ohadii*, which possesses the benefits of high biomass and valuable chemical product accumulation, but additionally holds scientific praise mainly for its exceptional photoprotection, native resistance to regular dehydration and extreme solar radiation, and record growth time (Treves et al., 2013, Treves et al., 2016; Ananyev et al., 2017). In large part, this exceptional ability to thrive in long periods of direct sunlight has been attributed to a mechanism of alternative photosynthetic electron flow, currently termed photosystem II-cyclic electron flow (PSII-CEF) (Ananyev et al., 2017; Steiner et al., 2025). The overall structure of the primary photosynthetic electron transport chain (PETC) protein, photosystem II (PSII), is vastly conserved throughout oxygenic phototrophs, with some variance in subunit structure depending on environmental conditions and stressors (Barber, 2003; Vinyard et al., 2013a; Shen, 2015; Oliver et al., 2023). An adaptable ability to modify the amounts of excitation energy delivered to the reaction center (RC) of PSII is of the utmost importance to organisms that survive in environments with dynamic light conditions. Typically, when a photon is captured by PSII, or higher energy light is captured and funneled to PSII by antenna, a charge separation event results in the transfer of the excited electron to the acceptor molecule plastoquinone (PQ) (Blomberg et al., 1998; Dekker and Van Grondelle, 2000; Renger and Schlodder, 2011; Ksas et al., 2022). However, this process is only possible if the primary PQ, Q_A_, is oxidized at the time of an attempted charge separation (Wasielewski et al., 1989; Broess et al., 2006; Garab, 2025). Conversely, if a build-up of reductant on the acceptor side of PSII is present during an attempted charge separation event and Q_A_ cannot be reduced, there arises a possibility for the excited chlorophyll to be further promoted to a long-lived triplet spin state that may interact with ubiquitous O_2_, creating reactive oxygen species (Santabarbara et al., 2002; Krieger-Liszkay, 2004; Pospíšil, 2012; Bhattacharjee et al., 2023). These oxygen radicals may indiscriminately interact with residues of PSII and cause irreparable damage, resulting in the grave energetic cost to the organism of replacing the protein supercomplex. This process of “photoinhibition” occurs increasingly at higher intensities of light (Aro et al., 1993; Cardona et al., 2012; Vass, 2012; Takeuchi et al., 2025), necessitating modifications to LEF in organisms subjected to dynamic light conditions, such as PSII-CEF.

While PSII-CEF is found in microalgae from various clades across the tree of life (Ananyev et al., 2016), *C. ohadii* is the most active known user of this mechanism, funneling over 90% of all charge-separated electrons into this pathway under its native light conditions (Ananyev et al., 2017). It is therefore critical to understand why this specific lineage developed such extraordinary photoprotection and whether its relatives have similar capabilities. Genome sequencing shows that *C. ohadii* is a close genetic relative of the aquatic microalga, *Chlorella sorokiniana* sp. UTEX 1663 (Kumar et al., 2014; Lizzul et al., 2018; Murik et al., 2024). *C. sorokiniana* has been shown to be tolerant of high light conditions (Kliphuis et al., 2011; Holdmann et al., 2018), but grows slower, sediments in liquid medium from starch body accumulation at lower optical densities, and is more photosensitive than *C. ohadii* (Treves et al., 2013; Murik et al., 2024). Despite these limitations, *C. sorokiniana* is a frequent subject of investigation for biomass and nutrient accumulation (Morita et al., 2000; Azaman et al., 2017; Yun et al., 2020). In an effort to characterize the effects of the minute genomic differences that have been established between these two species, we sought to characterize the photophysiology of both *Chlorella* at various light conditions as a direct experimental comparison. This work seeks to continue our comparative findings of *Chlorella ohadii* against low light adapted *Chlorella vulgaris* as a method of studying how PSII-CEF optimizes photosynthetic efficiency in the former (Steiner et al., 2025, Steiner et al., 2026). Our hope is that elucidating what physiologically differentiates *C. ohadii* from the related *C. sorokiniana* will result in potential advancements to biotechnical production of various phototrophic microbes.

## 2 Materials and methods

### 2.1 Culture growth

A CARON Model 7314–22 plant growth chamber at an internal temperature of 30 °C and atmosphere of 1.5% carbon dioxide housed each of the growing *Chlorella* cultures. Growth was initiated by low density inoculation (OD_730_ of 0.05) into 100 mL of BG-11 medium (Rippka et al., 1979), placed in autoclaved 250 mL Erlenmeyer flasks. Logarithmic growth time was determined by optical density at 730 nm (OD_730_). All reported testing herein was performed on cultures extracted during mid-log-phase growth and normalized to equivalent OD_730_, either by dilution in media, or concentration by centrifugation. Each *Chlorella* species was grown at both low and extreme light intensities, corresponding to photon deliveries of 20 µmol photons m^-2^ s^-1^ (low) and 2,000 µmol photons m^-2^ s^-1^ (extreme), supplied by a white light LED set to a 3500K spectrum. Extreme light grown cultures received humidified, filtered air from the atmosphere of the incubator bubbled at a rate of 0.3 L/min directly into their liquid medium to facilitate uninhibited growth. To ensure appropriate comparison of extreme light acclimated cultures, each species was acclimated to 2,000 µmol photons m^-2^ s^-1^ for a minimum of 40 generations before testing.

### 2.2 Fast repetition rate fluorometry

A glass cuvette (1 mm internal thickness), was used to suspend an aliquot of mid-log phase cells before a 180 s dark-adaptation, allowing decay to the dark-stable S-states of the water oxidizing complex, S_0_ and S_1_ (Mino and Kawamori, 2001). A modified Joliot-type spectrometer (JTS-150, SpectroLogiX) employed 24 μs laser pulses of approximately 480,000 µmol photons m^-2^ s^-1^ red light at a central wavelength of 636 nm. Each pulse worked to saturate the culture and advance each active water oxidizing complex by a single S-state. This pulse, dubbed a single turnover flash (STF), is repeated at a frequency of either 10 Hz or 50 Hz. In each instance, 15 groups of 50 STFs were recorded and averaged, with a 120 s wait time between each flash train to re-induce the dark stable population distribution (Ananyev and Dismukes, 2005; Gates et al., 2020). JTS-150 data was in turn processed by MATLAB r2023a and fitted by the model-dependent nonlinear least-squares matrix model VZAD (Vinyard et al., 2013b). The VZAD package used to average inefficiency parameters in this work was version 5.0, run using PyCharm 2024.2.1. Downstream measurement of PQ pool consumption was performed as in (Ananyev et al., 2026).

### 2.3 Molecular concentrations

Common use quantitative Western blotting procedures available from Agrisera were utilized for this work (Campbell et al., 2003; MacKenzie et al., 2005; Bouchard et al., 2006), with the addition of liquid nitrogen flash freezing in sodium dodecyl sulfate (SDS) based protein extraction buffer, proceeded by 5x 10 s on:10 s off cycles of ultra-fast tip sonication to perform total protein extractions. Bradford assay (Bradford, 1976) using Coomassie Blue protein reagent was utilized to quantify total protein concentrations. Suspended in 1x Laemmli buffer, 1.2 µg of total protein were loaded into individual wells of Bio-Rad precast polyacrylamide gels, alongside Thermo Scientific PageRuler protein ladders. SDS-page was run at 100 V for 50 minutes, and gels were transferred to a PVDF membrane by running an electro-transfer sandwich at 30 V for 40 minutes. Blots were inoculated in 1:10,000 primary antibody (PsbA protein, C-terminal, rabbit antibody: Agrisera product #AS05 084) and 1: 25,000 secondary antibody (goat anti-rabbit IgG horseradish peroxidase conjugate: Bio-Rad catalog #170-6515) for 1 hour each in 2% ECL blocking agent before treatment with a 50:50 peroxide:luminol mixture and imaging in a LI-COR Odyssey gel imager for 4 minutes. Pixel quantities used for PsbA concentrations were calculated in Fiji (n=3) against a PsbA standard curve (Agrisera product #AS01 016S) run alongside the testing blot. Final blot images against reference ladders are included as **Supplementary Figure S1**.

A Hansatech Oxygraph+ Clark-type electrode recorded oxygen production and consumption rates. Oxygen production rates were documented in the presence of corresponding growth light intensity. These rates were normalized to units of μmol O_2_ mg chl *a*^-1^ h^-1^ via chlorophyll extraction, determined according to the extinction coefficients for chlorophyll extraction in chilled methanol established in (Porra and Thompson, 1989). Chlorophyll measurements were averaged from both biological and technical triplicate (n=9). Protein extraction, rate oximetry and chlorophyll extraction testing cultures were all concentrated to equal cell density of OD_730_ 0.60 before testing. Average time between charge separations per PSII has been quantified by converting oxygen production to O_2_ s^-1^ PsbA^-1^ and integrating four charge separations per O_2_ created. At 1 PsbA per PSII, this number results in the quantity of charge separations per PSII per second.

### 2.4 Q_A_^-^ reoxidation kinetics

Following the protocol of (Gorbunov et al., 1999), decaying background fluorescence over time was recorded at increasing dark intervals, starting at 5.4 µs and ending at 4.1 ms. Best fit values of background fluorescence were computationally determined by derivation, and individual F_v_/F_m_ values for each STF were plotted against their respective dark period length in μs using an average of biological triplicate datasets (n=3). Using Origin 2023b, decay regression analysis fitted the decay parameters used in the determination of Q_A_^-^ oxidation amplitude. Q_A_^-^ reoxidation testing cultures were diluted to an OD_730_ of 0.10 to ensure complete initial reduction of plastoquinone at the Q_A_ binding site that is reported as 100% reduction at time point zero.

### 2.5 Kinetic studies via redox changes

Various kinetic processes within the photosynthetic apparatus can be monitored by the JTS-150 using an illumination window of actinic light at 630 nm to promote photosynthetic electron transport chain (PETC) operation while LEDs tuned to distinct spectral wavelengths record redox changes in PETC components. Herein, these include the reduced forms of cytochrome (cyt) *b_6_f* components (cyt *b_6_* and cyt *f*), the oxidized form of plastocyanin (PC), the reduced form of P700, and the proton motive force via electrochromic shift (ECS). Descriptions of the hardware setup applied are more explicitly detailed in (Steiner et al., 2025), but will be summarized below. For each test, cultures were concentrated to an OD_730_ of 2.00 and the instrument was balanced to 6.5 V. Reported data are averages of biological triplicate measurements (n = 3).

#### 2.5.1 Cytochrome *b_6_f* and plastocyanin redox kinetics

3,200 µmol photons m^-2^ s^-1^ actinic light was applied for a total of five seconds, while reduced cyt *b_6_* (574 nm), reduced cyt *f* (563 nm), and oxidized PC (554 nm) were monitored, with a baseline corrective measurement at 546 nm. A BG-39 multiple bandpass filter was placed in the beam path.

#### 2.5.2 Photosystem I (P700) kinetics

1,300 µmol photons m^-2^ s^-1^ actinic light was applied for a total of 25 seconds, while reduced P700 was monitored at 810 nm. A P700 multiple bandpass filter was placed within the beam path.

#### 2.5.3 Electrochromic shift

1,200 µmol photons m^-2^ s^-1^ actinic light was applied for a total of five seconds, while ECS was monitored at 520 nm, with a corrective measurement for light scattering through the thylakoid at 546 nm. These wavelengths were determined previously by diffused-optics flash spectroscopy (Kramer and Sacksteder, 1998). A BG-39 multiple bandpass filter was placed within the beam path.

### 2.6 77K spectrofluorometry

750 µL of testing culture was flash frozen by plunging a 7” medium wall glass NMR tube into liquid nitrogen. The frozen tubes were then placed in a JASCO FP-8300 spectrofluorometer, where chlorophyll *a* fluorescence emission spectra were first induced with an excitation wavelength of 435 nm by a continuous xenon lamp. Subsequently, carotenoid spectra were recorded proceeding an excitation wavelength of 488 nm (Lamb et al., 2018). All spectra were concentrated to an OD_730_ of 0.60 prior to testing collected at a resolution speed of 500 nm/min. Each test is a representative average of biological triplicate measurements (n = 3) and error provided is standard deviation of those measurements, obtained using OriginLab 2023b. The Peak Pick analysis function in OriginLab 2023b was used for Gaussian deconvolution of the 77K spectra. This method was employed as a means of quantifying relative abundance of chlorophyll *a* and carotenoid by each photosystem within either *Chlorella* species.

## 3 Results

### 3.1 Capabilities of photosystem II photochemistry

One of the most frequently observed physiological differences between these closely related *Chlorella* is the higher amount of photobleaching that occurs in *C. sorokiniana*. Originally, when *C. ohadii* was isolated and the photoprotective capabilities of the two species were directly compared, *C. sorokiniana* was almost entirely bleached after a 24 hour extreme light illumination period (3,500 µmol photons m^-2^ s^-1^ in this case) (Treves et al., 2013). *C. ohadii* also maintained a steady PsbA content over the entire period and showed no change in overall PSII operation when presented with the antibiotic chloramphenicol, while *C. sorokiniana* was almost fully inhibited by the antibiotic after 6 hours of illumination. Interestingly, over this photoperiod, the oxygen production of *C. ohadii* increased, while it declined in *C. sorokiniana*. Clearly there are differences to the regulation of photochemistry in PSII between these organisms that warrant further investigation. After acclimating *C. sorokiniana* to extreme light conditions (12:12 h diurnal cycle at an illumination of a constant 2,000 µmol photons m^-2^ s^-1^, representative of approximately irradiance received in direct sunlight) growth rates were recorded and fast repetition rate fluorometry (FRR) was used to monitor PSII operation in both low light (LL; 20 µmol photons m^-2^ s^-1^) and extreme light (EL) grown cultures of either *Chlorella* species. During log-phase growth, the EL grown cultures exhibited an average (n=3) minimum doubling time under 2 hours, 1.5 hours for *C. ohadii* and 1.8 hours for *C. sorokiniana*, while this value for both species grown at LL was 3.9 days. EL *C. sorokiniana* possessed a slight paleness in its green coloration during log-phase growth in comparison to the other three conditions, which disappeared once the culture entered the stationary growth phase.

**Figure 1:**
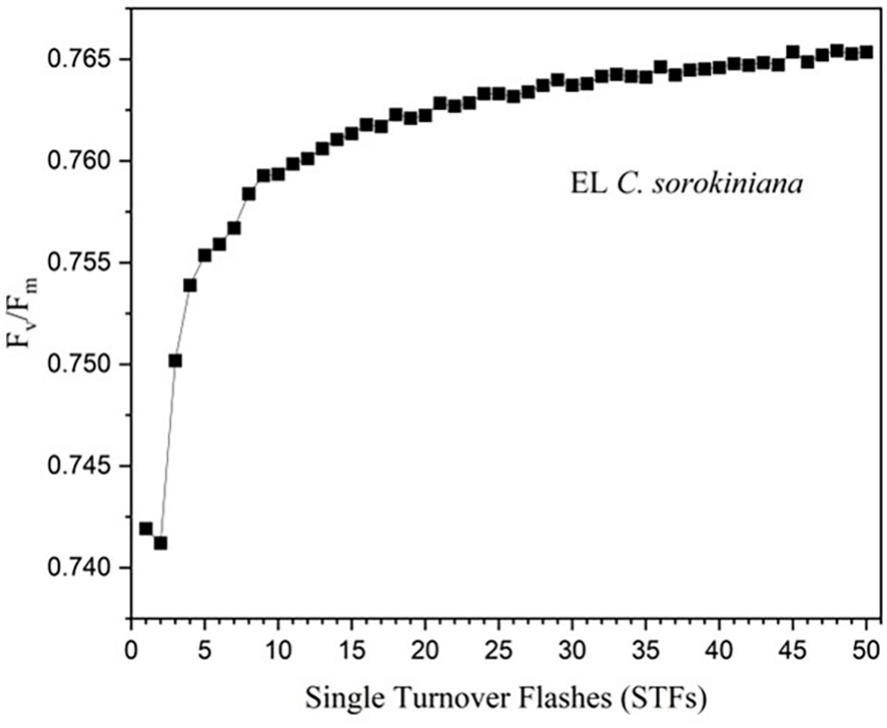
Initial trial of extreme light acclimated *Chlorella sorokiniana* fast repetition rate fluorometry. F_v_/F_m_ over 50 single turnover flashes in extreme light grown *C. sorokiniana*.

**Figure 2:**
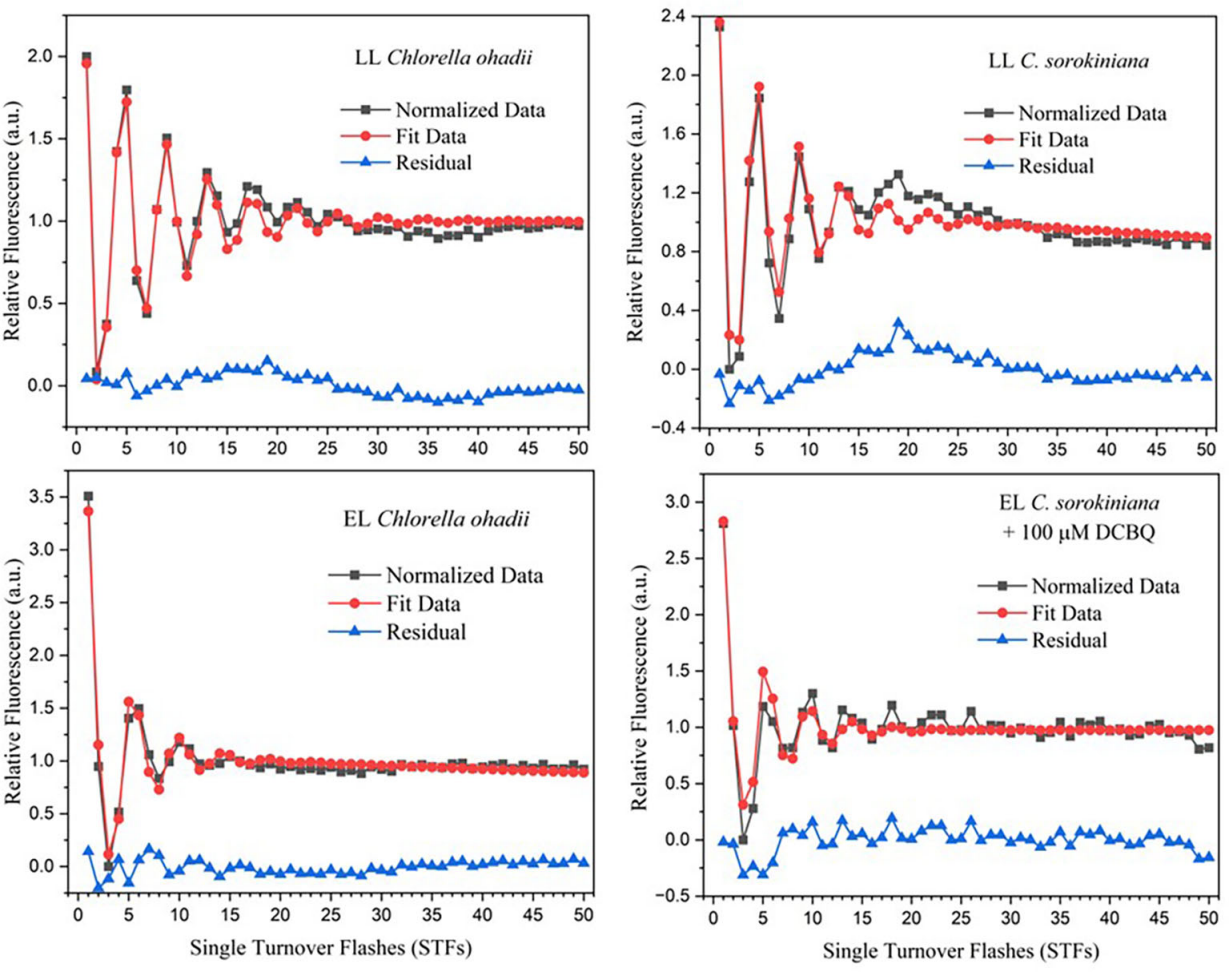
Fast repetition rate fluorometry of *Chlorella ohadii* vs. *Chlorella sorokiniana* across light conditions. Raw 10 Hz fast repetition rate fluorometry values are normalized (black trace) and fit (red trace) in **(A)** LL grown *C. ohadii*, normalized to an F_v_/F_m_ value of 0.668 **(B)** LL grown *C. sorokiniana*, normalized to an F_v_/F_m_ value of 0.717 **(C)** EL grown *C. ohadii*, normalized to an F_v_/F_m_ value of 0.829 and **(D)** EL grown *C. sorokiniana*, with addition of 100 μM DCBQ, normalized to an F_v_/F_m_ value of 0.787.

When monitoring the variable fluorescence of the PSII RCs in EL *C. sorokiniana* by a series of single turnover flashes (STFs) at a frequency of 10 Hz, what appears is a rising kinetic curve with no oscillatory behavior from the donor side operation of PSII (**Figure 1**). In an effort to achieve visibility of the oscillatory behavior by the water oxidizing complexes (WOCs) in this EL culture, the introduction of an exogenous quinone was used as a PQ mimetic, increasing linear electron flow. Initially, an attempt was made to rescue WOC oscillation by adding 2,5-dimethyl-1,4-benzoquinone as an exogenous electron acceptor (not shown), however there was again a rising variable fluorescence kinetic present, similar to that in **Figure 1**. Subsequently, an effective comparison of the WOC operation of EL acclimated *C. sorokiniana* was established with the addition of 100 µM 2,6-dichloro-*p*-benzoquinone (DCBQ), and is reported alongside control measurements of either *Chlorella* species from LL and EL growth conditions in **Figure 2**. For comparison, FRR of EL grown *C. ohadii* with exogenous quinone added is available in **Supplementary Figure S2**, but every case of adding quinone to *Chlorella ohadii* grown in its natural light condition diminishes WOC oscillation quality.

With the *C. sorokiniana* grown in LL conditions (**Figure 2B**), there are multiple visible oscillations in the RC variable fluorescence. Here, one unique feature that has not been observed in the LL grown *C. ohadii* (**Figure 2A**) is a sigmoidal feature after approximately five full progressions of the Kok cycle (20 STFs). When the inefficiency parameters of these FRR traces were calculated using the nonlinear matrix model VZAD (**Table 1**), there is a discrepancy that appears between the LL conditions in the parameter epsilon (state inactivation), which disappears from the *C. sorokiniana* culture at EL when excess DCBQ is added. Between the remaining inefficiency parameters (alpha, beta, and delta) there is approximately equal quality of WOC operation between the LL *Chlorella*.

**Table 1:** Donor side cycling of *Chlorella ohadii* vs. *Chlorella sorokiniana*. Water oxidation inefficiency parameters, calculated from the 10 Hz flash rate fast repetition rate fluorometry detailed in **Figure 2**.

Inefficiency parameter	LL *C. ohadii*	EL *C. ohadii*	LL *C. sorokiniana* ​	EL *C. sorokiniana* + 100 µM DCBQ ​
Alpha (miss)	0.111	0.171​	0.099​	0.146
Beta (double hit)	0.017	0.045​	0​	0.060
Delta (backward transition)	0	0.015​	0.017​	0
Epsilon (state inactivation)	0	0​	0.085​	0
Quality Factor (α+β+δ+ϵ)-1	7.82	4.33​	4.99​	4.85​

**Figure 3:**
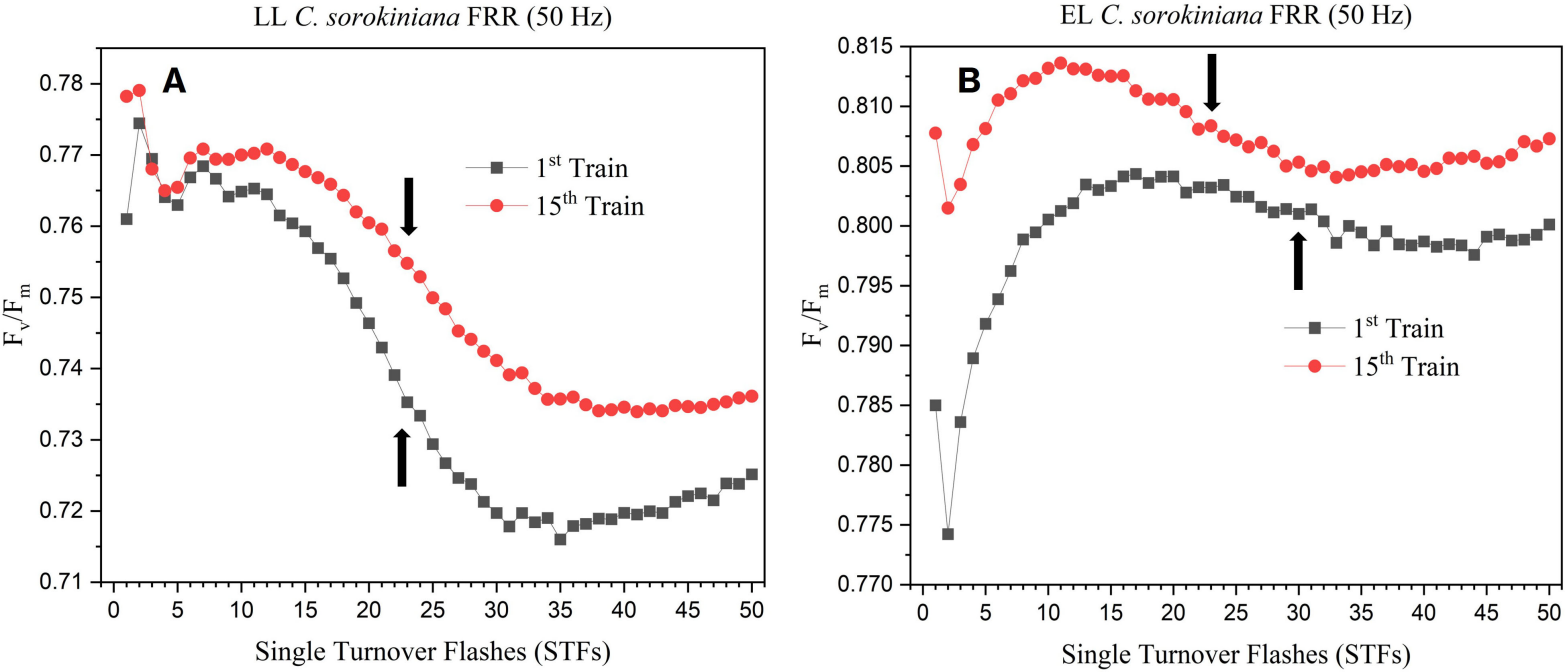
High frequency flash probing of *Chlorella sorokiniana*. FRR traces of the first (black trace) and last (red trace) flash trains monitoring **(A)** LL grown and **(B)** EL grown *C. sorokiniana*, run at an STF frequency of 50 Hz, with midpoints of the sigmoidal shape indicated by black arrows.

To further monitor the effects occurring by limitations in the PQ pool, we have also reported the FRR traces of *C. sorokiniana* run at a higher STF frequency of 50 Hz (**Figure 3**) to see how quickly this species runs out of available PQ. This 20 ms flash rate is slower than rate limitation of electron transfer from the WOC, but should effectively limit the continuous turnover of the PQ pool into the Q_B_ site by diffusion. Comparisons of flash trains (50 STFs) at 50 Hz over the course of time also may illustrate some important structural changes in the PETC architecture, as can be seen between the first and last flash trains illustrated in **Figure 3**. F_v_/F_m_ is conventionally considered a means of quantifying quantum efficiency of PSII, with its decline a show of abiotic stress, typically used to relate quantities of non-photochemical quenching (NPQ) and photoinhibition. In both growth light intensities, the observed variable fluorescence in **Figure 3** is higher in the final train (red trace) than during the first train (black trace).

There are some important differences in the shape of these fluorescence curves. First, the amplitude of the LL *C. sorokiniana* curves reach a lower point by the end of any flash train, where the EL grown *C. sorokiniana* reaches an increased photochemical efficiency. This difference may owe to cyclic electron transfer in the EL culture, relieving reductant at the acceptor side of PSII while the LL culture cannot achieve this. Second, each of these curves has a (reverse) sigmoidal shape, indicative of depleting the available PQ pool. The end of this sigmoidal feature has been shown to relate to the end of electron transfer from Q_A_, offering an approximation of the PQ: PSII ratio in the cells by the mid-point of the curve (indicated by arrows), as each PQ molecule accepts two electrons before exchanging from the site. The approximated PQ pool size of the LL *C. sorokiniana* would then be 22 PQ: PSII in each flash train. In the EL culture, the available and oxidized PQ is approximately 30 PQ: PSII in the first train, a significant increase. Interestingly, this decreases to 22 by the 15th train. We have proposed previously that acclimation to the EL conditions these *Chlorella* are subjected to increases the size of the PQ to help modulate PSII-CEF. It appears that this PQ pool also stays progressively reduced during repeated light:dark periods, potentially to remain primed for cyclic electron transfer once illumination returns. Further analysis of PSII operation was evaluated by quantum yield analysis (**Table 2**).

**Table 2:** Chlorophyll and protein content in *Chlorella ohadii* and *Chlorella sorokiniana*.

Parameter	LL *C. ohadii*	EL *C. ohadii*	LL *C. sorokiniana* ​	EL *C. sorokiniana* ​
[chl a] (μg/mL)​	7.40 ± 0.36​	2.86 ± 0.11​	7.23 ± 0.36​	3.68 ± 0.34​
[chl b] (μg/mL)​	1.89 ± 0.26​	0.43 ± 0.13​	1.83 ± 0.10​	0.34 ± 0.10​
Total Protein (μg/mL)​	810 ± 10	826 ± 4	813 ± 3	823 ± 8
[PsbA] (pmol/mL)​	51.4 ± 3.4	53.8 ± 1.4​	33.4 ± 0.7	66.2 ± 2.7
O_2_ Evolution ​ (μmol O_2_ mg chl a-1 h-1)​	44.2​ ± 2.2	1,250​ ± 50	42.5​ ± 2.2	1,210​ ± 80
Average Time Between Charge Separations per PSII (ms)​	141 ± 2.2	13.5​ ± 0.8	97.8​ ± 6.8	13.4 ± 1.6

Chlorophyll, PsbA, and protein concentrations, alongside PSII operation kinetics in the two closely related *Chlorella*. Chlorophyll extraction quantities are averages of n=3 biological replicates from each culture, solubilized in chilled methanol. PsbA protein quantities were obtained by Western blot. Blot images used for quantification are available in **Supplementary Figure 1**. Values of chlorophyll, total protein, and PsbA presented are the average of at least n=3, ± standard deviation. The remaining quantities are averages of at least n=3, with uncertainties based on chlorophyll or PsbA quantities.

The change in chlorophyll *a* concentration across growth light conditions is more pronounced in *C. ohadii* comparatively. At EL conditions, the chlorophyll *a*:*b* ratio is significantly higher in *C. sorokiniana* than *C. ohadii*, at 10.8 and 6.65, respectively. Literature of *C. ohadii* has shown high chlorophyll *a*:*b* ratios, closer to what is observed here with *C. sorokiniana*. This lower ratio, suggesting a larger association of LHC than expected from literature suggests an even greater minimization to photoinhibition than has been seen previously. Increasing from low to extreme light growth, *C. ohadii* exhibits only a 4.7% difference in PsbA concentration, illustrating a conservation of intact and active photosystem II, while *C. sorokiniana* changes its PsbA expression by 98%. It appears that *C. ohadii* maintains healthy PSII regardless of the light condition, while *C. sorokiniana* overexpresses large amounts of PsbA to replace them as they are photodamaged. Despite these differences between the PSII antenna associations, and overexpression of PsbA in EL conditions, both species have very similar normalized oxygen evolution and average charge separation times. Regarding the kinetics of the EL *Chlorella* reported in **Table 2**, we observed a plateaued average time between charge separation per PSII of ~13–14 ms, suggesting that this is the upper limit achievable in what are currently some of the fastest growing phototrophs on record.

### 3.2 Pigment distributions

**Figure 4:**
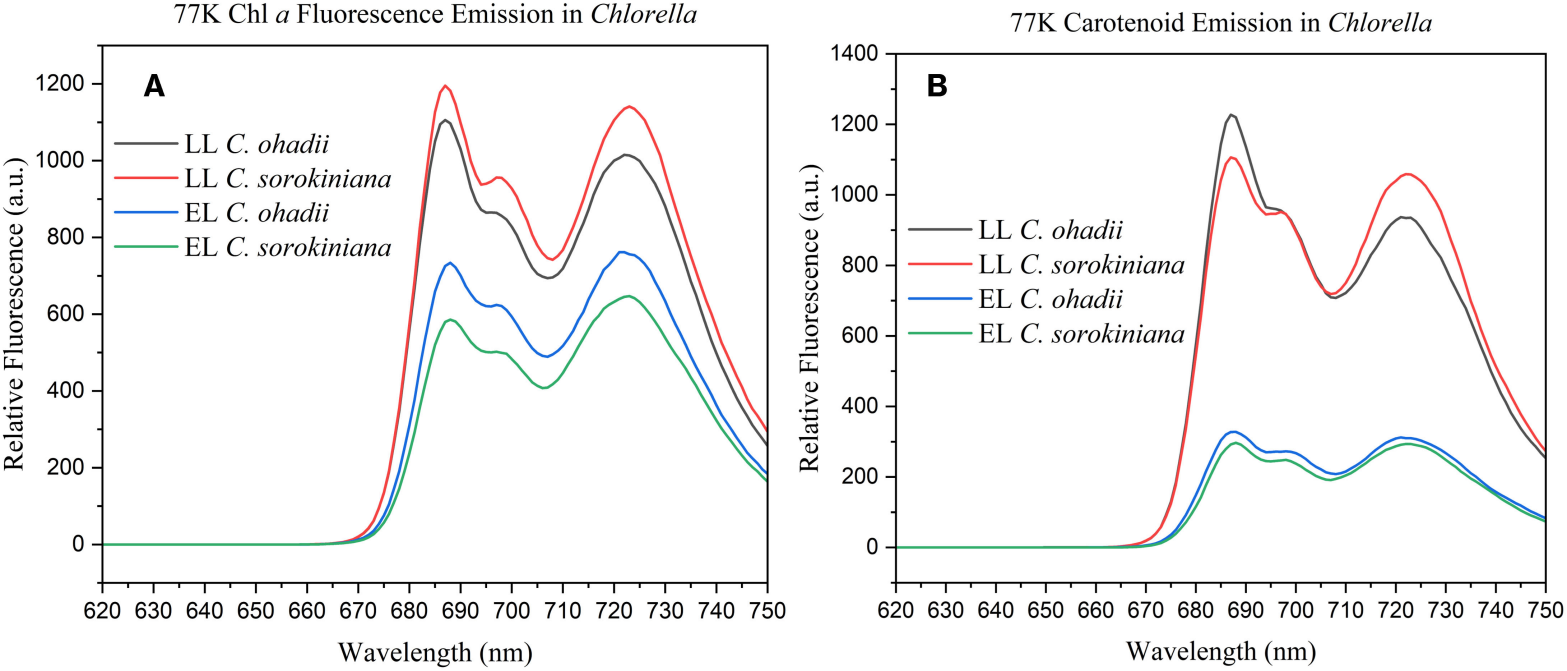
Normalized 77K spectra of pigments in *Chlorella ohadii* vs. *Chlorella sorokiniana*. Cryogenic fluorescence emission spectra of whole cell *Chlorella* cultures flash frozen in liquid nitrogen. **(A)** Chlorophyll *a* emission spectra produced by continuous excitation with 435 nm light. **(B)** Carotenoid pigment fluorescence spectra from continuous excitation with 488 nm light. In either panel, LL *C. ohadii* is represented by the black trace, LL *C. sorokiniana* by the red trace, EL *C. ohadii* by the blue trace, and EL *C. sorokiniana* by the green trace.

Pigment distribution within the PETC is greatly descriptive in terms of the energetic needs of a phototroph. Utilizing cryogenic fluorescence spectroscopy at 77K is an advantageous and non-invasive way to monitor relative light pigment association with both photosystems, as well as quantifying a comparison of PSI and PSII in whole cell cultures. With this method, amplitudes and electromagnetic shifting of reaction center pigments can be compared between species. Normalized 77K fluorescence reaction center emission spectra (**Figure 4**) by excitation of chlorophyll *a* and carotenoids show slight differences in the quantities of chlorophyll *a*, but fairly consistent carotenoid content between the two species. Interestingly, if the amplitudes of chlorophyll *a* emission are slightly adjusted, the spectra show almost complete overlap (**Supplementary Figure S3**). The unscaled 77K spectra in **Figure 4** have been fit by Gaussian analysis (**Table 3**) to make direct comparisons of pigment associations on a per OD_730_ basis.

**Table 3:** Gaussian areas of Chlorella ohadii and Chlorella sorokiniana.

Parameter	LL *C. ohadii*	EL *C. ohadii*	LL *C. sorokiniana* ​	EL *C. sorokiniana*​
F685 Area	14,300 ± 94 ​	9,180 ± 160 ​	15,100 ± 145​	7,760 ± 68​
F695 Area	7,100 ± 150 ​	5,620 ± 260 ​	9,360 ± 251​	3,400 ± 126​
F685: F695	2.01 ± 0.04 ​	1.63 ± 0.08 ​	1.62 ± 0.05​	2.28 ± 0.09​
PSI Complex Area	37,400 ± 420 ​	26,200 ± 740 ​	39,400 ± 713​	23,500 ± 1140​
PSII:PSI	0.571 ± 0.008 ​	0.565 ± 0.020 ​	0.622 ± 0.013​	0.474 ± 0.024​

Areas and ratios of chlorophyll *a* fluorescence, obtained from Gaussian fitting of the spectra in **Figure 4a**. F_685_ represents active chlorophyll reaction centers of PSII, combining LHCII, CP47, and CP43 emission. F_695_ is attributed to chlorophyll trap emission in CP47. Photosystem I components are centered around 725 nm. Values presented are fit values of averaged traces from at least n=3, ± reported standard error.

The most drastic inter-species discrepancy in pigment association is the change to the PSII:PSI ratio in increasing light conditions. While this ratio does not change much in *C. ohadii*, *C. sorokiniana* has a much larger loss of active PSII reaction centers. Combined with the comparatively higher PsbA content in *C. sorokiniana* per **Table 2**, it appears as though this species creates excess amounts of PsbA that are inactive and waiting for replacement, unlike what is seen in *C. ohadii*. This corroborates that findings of FRR and earlier literature, that despite their large genetic overlap, one glaring difference between these species is that *C. ohadii* exhibits far more exceptional photoprotective ability at extreme light.

### 3.3 Q_A_^-^ reoxidation kinetics

**Figure 5:**
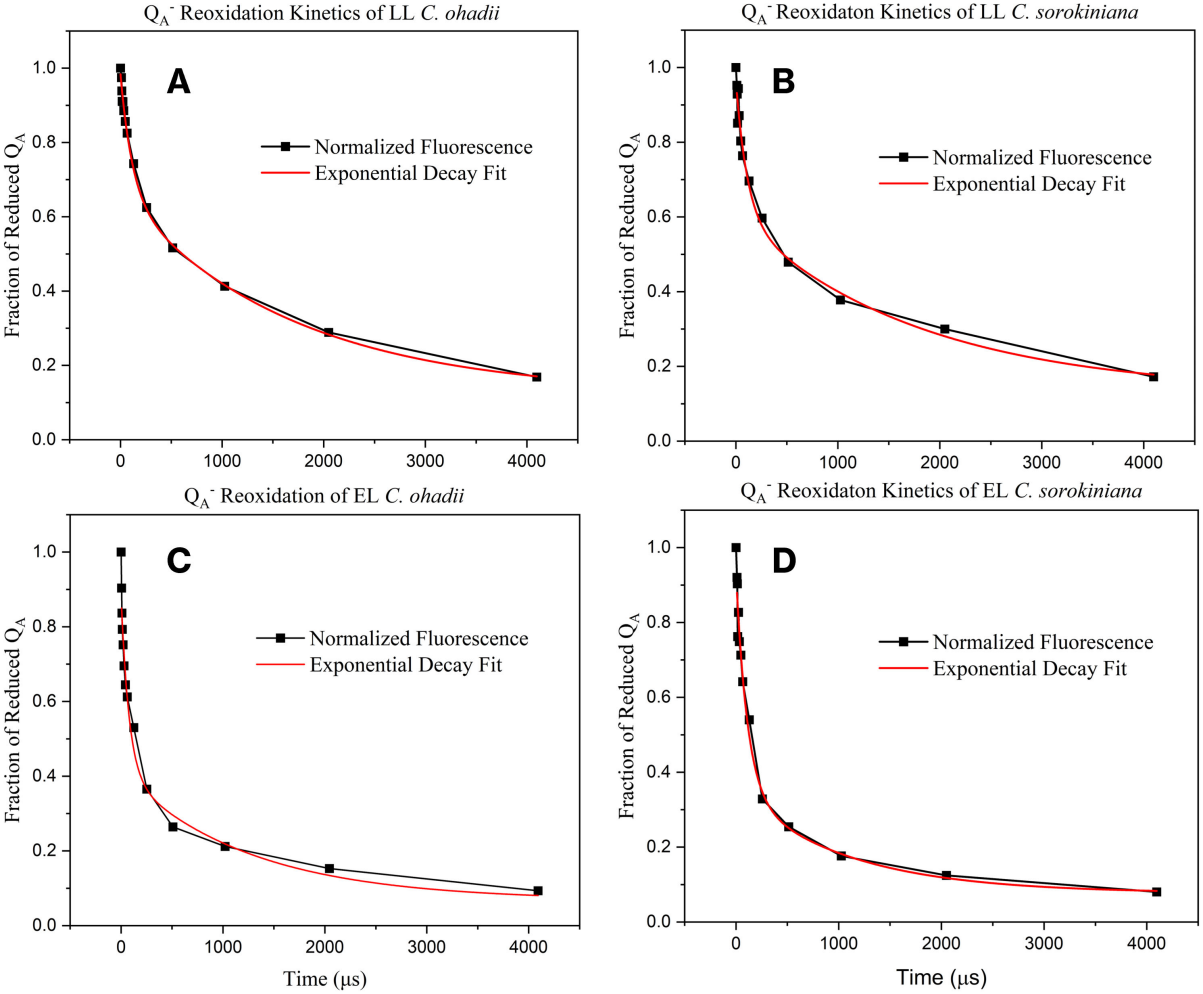
Decay curves of Q_A_ reduction in closely related *Chlorella*. Kinetics of Q_A_^-^ reoxidation, monitored by quenching of RC fluorescence following a fully saturating light pulse at time point zero. Dynamic background fluorescence at each proceeding time point (F_n_) was normalized (black trace) to the overall fluorescence maximum (F_m_) and fit (red trace) to a two-phase decay kinetic to obtain rates of primary (t_1_) and secondary (t_2_) electron transfer from Q_A_^-^. Panels illustrate the decay fits for **(A)** LL *C. ohadii*, **(B)** LL *C. sorokiniana*, **(C)** EL *C. ohadii*, and **(D)** EL *C. sorokiniana*.

Moving to the regulation of electron transfer out of PSII, examination of the PQ pool redox poise is integral to determining variances in the operation of the PETC and PSII-CEF in individual species, as this poise regulates rates of linear electron flow, proton gradient production, and the need for electron recycling. The kinetics regarding the reduction of mobile PQ in the thylakoid (at the Q_B_ binding pocket) are of special importance. To observe the redox kinetics of the PQ pool, the decay of reduced Q_A_ was fit to a biphasic regression analysis. These fits for each *Chlorella* species in either light condition are illustrated in **Figure 5**, with output of kinetic parameters for each fit listed in **Table 4**. Visibly, there is a larger disparity in the amplitudes of the fast and slow phases of electron transfer (A_1_ and A_2_, respectively) in the EL cultures when compared to LL. Extreme light (and assumedly high PSII-CEF) increases the population of centers performing primary (fast) electron transfer and slows the kinetics of the secondary electron transfer. The population of inactive centers (y_0_), with either fully reduced or unbound PQ, is minimized in each EL culture, detailing a near complete use and reduction of the PQ pool. This population is smallest in EL grown *C. ohadii* (7.6%). While these trends in quick and vast removal of electrons from Q_A_ appear in both *Chlorella* species when comparing the extreme light Q_A_^-^ reoxidation to that of the low light, it is important to note that the decay curves in **Figure 5** do not differentiate between forward transfer and backward transfer (charge recombination). As shown in **Figure 1**, recombination could act as a major contributor to the fast decay in any of these traces, but is likely in the LL acclimated cultures, as they are poised to funnel as much light as possible to the RC, but not necessarily to properly utilize it.

**Table 4:** Various acceptor side populations in *Chlorella*.

Parameter	LL *C. ohadii*	EL *C. ohadii*	LL *C. sorokiniana*​	EL *C. sorokiniana*​
y0	12.5 ± 0.3%​​	7.6 ± 0.3%	13.4 ± 0.9%​	8.2 ± 0.5%​
t1	117 ± 2 µs	77.4 ± 2.1 µs	112 ± 5 µs​	110 ± 4 µs​
A1	32.8 ± 1.3%​​	53.7 ± 0.6%	37.6 ± 0.8%​	62.7 ± 1.3%​
t2	1.69 ± 0.03 ms​​	1.25 ± 0.05 ms	1.80 ± 0.11 ms​	1.08 ± 0.10 ms​
A2	54.7 ± 2.8%​​	38.7 ± 0.6%	49.0 ± 0.7%​	29.1 ± 1.2%​

Parameters of photosystem II acceptor side electron transfer kinetics, retrieved from analysis of the decay traces in **Figure 5**. Values presented are fit values of averaged traces from at least n=3, ± reported standard error.

### 3.4 Other PETC processes

**Figure 6:**
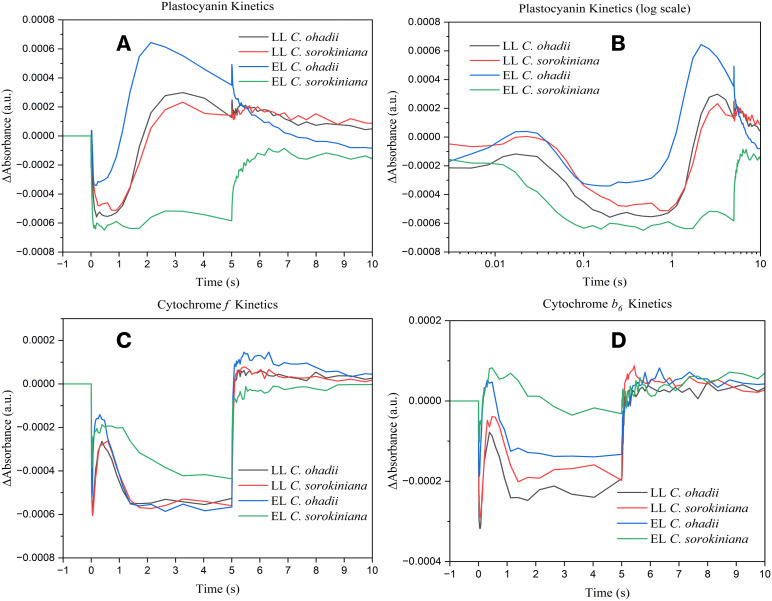
Cytochrome *b_6_f* Kinetics in *Chlorella ohadii* and *Chlorella sorokiniana*. Kinetics of the cyt *b_6_f* protein complex components. Absorbance changes in the oxidized form of PC during a five second actinic illumination period are reported in both **(A)** linear and **(B)** logarithmic time scales for whole cell *Chlorella* cultures. Absorbance changes due to the reduced forms of **(C)** cyt *b_6_* and **(D)** cyt *f* are reported to monitor Q-cycle and linear transfer through cyt *b_6_f*, respectively.

**Figure 7:**
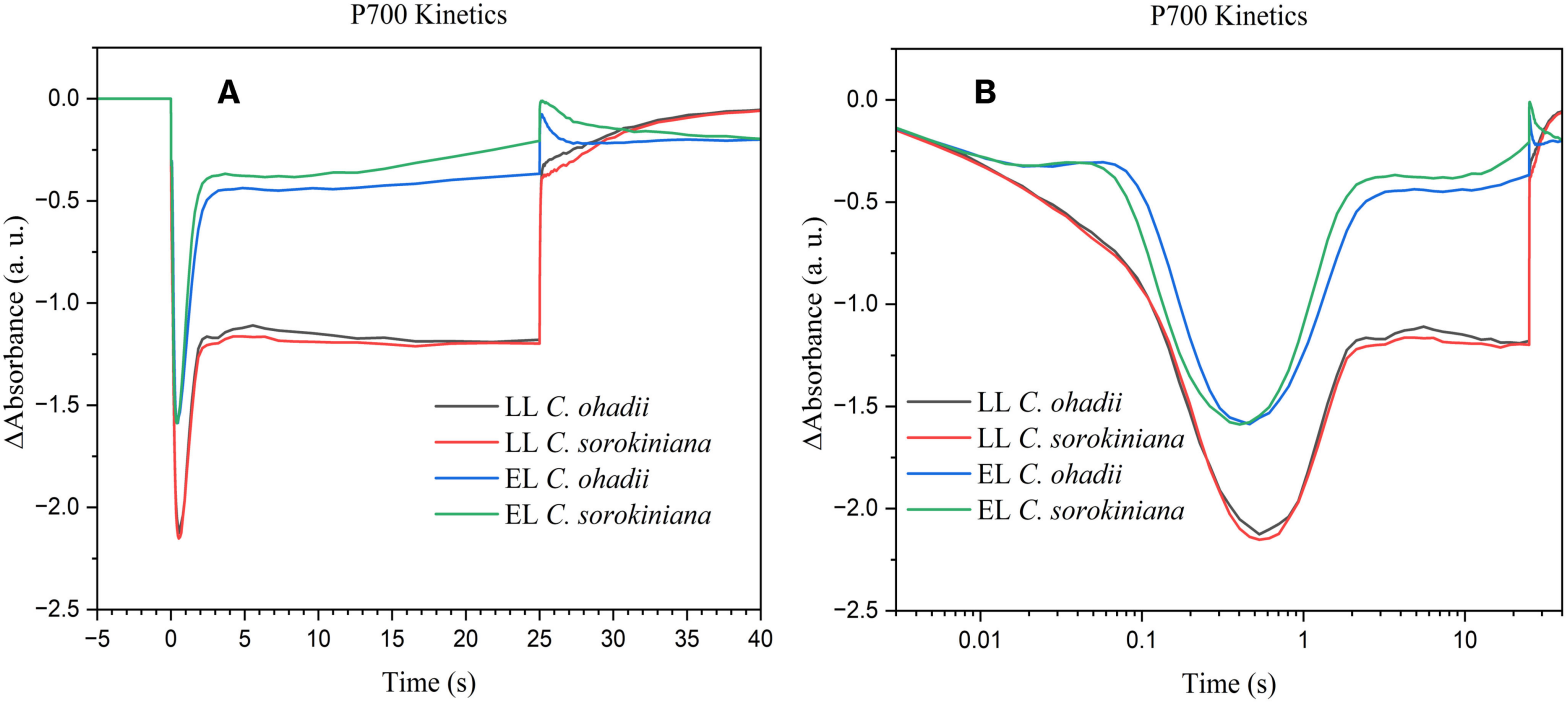
Photosystem I redox changes in closely related *Chlorella*. Redox kinetics of the PSI reaction center, P700, measured by absorbance changes at 810 nm when presented with 25 seconds of actinic illumination, in both **(A)** linear and **(B)** logarithmic timescales. In either panel, LL *C. ohadii* (black trace), LL *C. sorokiniana* (red trace), EL *C. ohadii* (blue trace), and EL *C. sorokiniana* (green trace) are all representative averages, normalized to OD_730_.

Perhaps the largest differentiation in the way these two *Chlorella* species perform electron transfer through their PETC is in the operation of the cyt *b_6_f* complex (**Figure 6**). While both LL grown cultures are virtually identical in the way that most other PETC components are, EL grown *C. ohadii* and *C. sorokiniana* exhbit drastically different redox poise of their PC, cyt *f*, and cyt *b_6_*. Surprisingly, *C. sorokiniana* seems to have a much higher reduction of all three of these components, meaning that there is higher electron tranfer through this protein, and an increase Q-cycle operation in this species. EL *C. sorokiniana* also has the fastest intial reduction of its PC pool. This large discrepancy is intriguing, considering the fairly consistent result in Q_A_^-^ reoxidation kinectics between the two EL cultures listed in **Table 4**. One potential modification to the PETC that could cause a largely reduced PC pool would be the operation of photosystem I (PSI) cyclic elecron flow returning reductant to the cyt *b_6_f* complex. In order to monitor this, PSI operation kinetics by absorbance measeurement are reported in **Figure 7**.

*C. ohadii* and *C. sorokiniana* would appear to have the same relative amplitude of PSI operation when grown in equivalent light conditions. The traces for the LL conditions (black and red) are indistinguishable from each other, while there are some minute differences in the EL traces (blue and green). First, the PSI in *C. sorokiniana* appears to start operating several ms earlier than *C. ohadii*. Both traces reach the same minimum (showing a very similar quantity of active PSI, illustrated in **Figure 7**), but there is a slightly higher reduction to the population of PSI in *C. sorokiniana*, which slowly becomes more reduced over time. In earlier work on EL *C. ohadii*, we observed a tandem operation of cyclic electron flow around both photosystems; this appears to be another example of this tethering, with the amplitude of PSI-CEF appearing slightly higher in *C. sorokiniana*.

**Figure 8:**
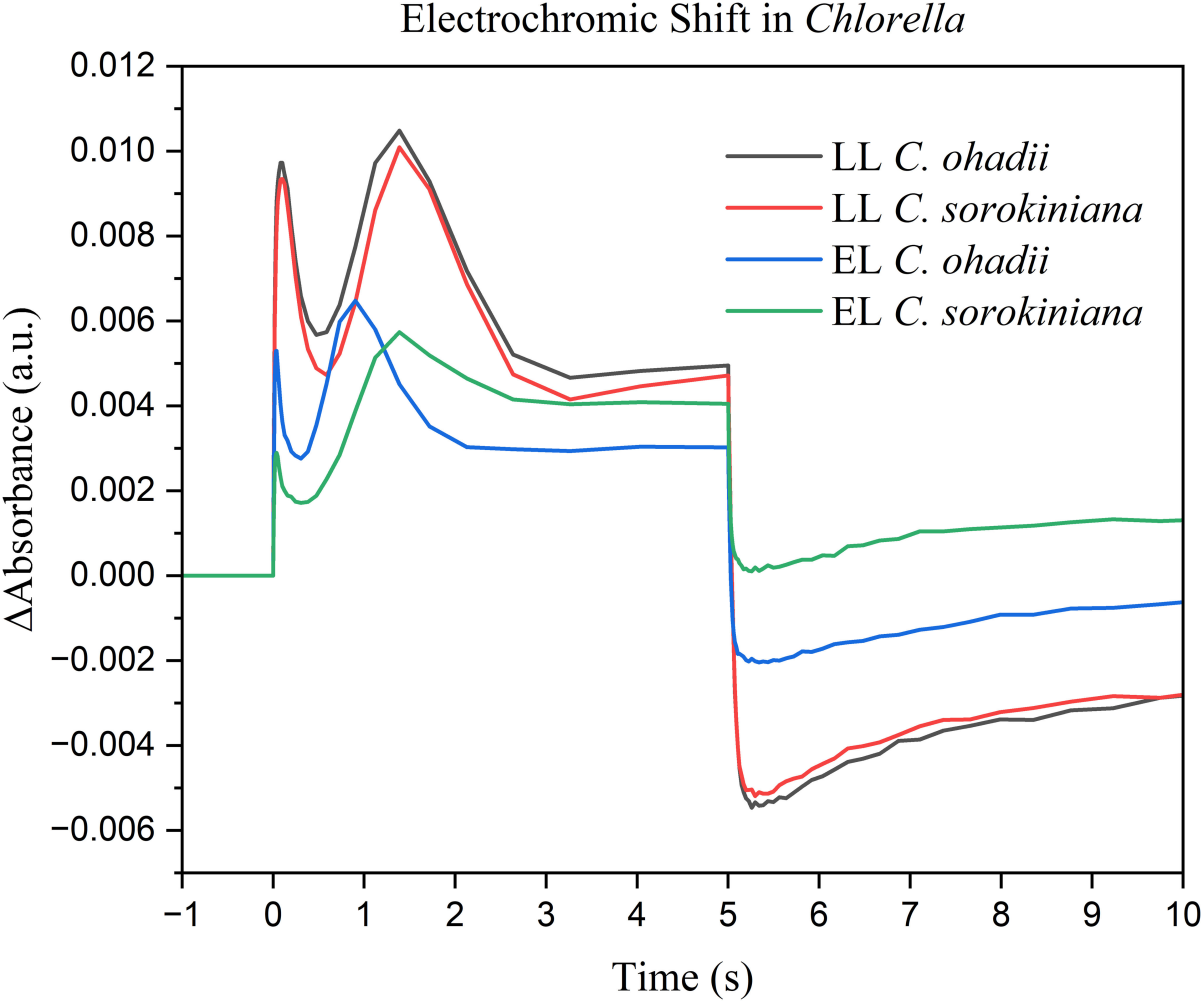
ECS kinetics of *Chlorella ohadii* vs. *Chlorella sorokiniana*. ECS measurements in a five second actinic illumination, used to monitor the changes in proton motive force components. Positive change is attributed to a larger gradient across the membrane compared to the dark baseline (zero absorbance).

Holistically looking at the consequences of these differences, electrochromic shift (ECS) (**Figure 8**) was utilized to measure the relative proton motive force (pmf) in these organisms to compare the ATP output from the PETC. Once again, there is no significant distinction in the operation of the LL cultures, and on a per OD_730_ basis, it appears that the operation of the PETC is virtually identical in these cultures. Both EL cultures have a smaller contribution to the electrochromic bandshift than the LL *Chlorella*, as the EL growth minimizes the photosystem quantities in each species. This results in a pmf closer to the dark baseline throughout the entire illumination period. There is also an expedited start of both ATP synthesis and carbon fixation in the EL *C. ohadii* compared to any of the other three traces. This suggests that there is a faster onset of PSII-CEF in native (EL) *C. ohadii* curve, keeping the gradient closer to the dark baseline. This major discrepancy would work to explain the contrast in photoprotective abilities between the two species if *C. sorokiniana* is not mechanistically equipped to perform high levels of PSII-CEF and is instead prioritizing PSI-CEF, contributing to a comparatively larger, and delayed equilibration of, the pmf.

## 4 Discussion

This spectroscopic overview of the physiology of *C. ohadii* against *C. sorokiniana* works towards understanding the unique nature of *C. ohadii* and how it is differentiated from its closest relative. It is worth noting that the culture of EL *C. ohadii* reported here has been lab acclimated for several years, and inoculated for over 100 generations. The *C. ohadii* our group examined herein may be better acclimated to continuous extreme light than that which was originally isolated. Likewise, the *C. sorokiniana* cultures used for the data reported here have also been acclimated to EL for a minimum of 40 generations to work towards conserved physiological acclimations. By our battery of testing, the two LL grown species are virtually indistinguishable in any operative form of their PETC, with statistically equivalent WOC operation, distribution and quantity of photosystems, pmf, and quantum yields. At extreme light conditions however, *C. ohadii* continues to be unmatched in its robust nature and overall photosynthetic ability. Conversely, *C. sorokiniana*, while tolerant and quickly replicating at EL, seems to moderate this condition differentially, employing PSI-CEF to optimize ATP production and retain more reductant downstream of PSII, while saving a reserve pool of extra chlorophyll *a* as inactive PsbA and potentially in other photosystem proteins.

It has been postulated that chloro-substituted benzoquinones work more effectively than methyl or methoxy-substituted quinones at acceptance of electrons from PSII (Dekker et al., 1984; Graan and Ort, 1986; Fu et al., 2017; Kamada et al., 2023). This effect is likely species specific, owing to alterations to the Q_B_ site composition (Ananyev et al., 2016; Jugulam et al., 2025), which may even be altered by dynamic light conditions. This clade, and particularly *C. ohadii*, is already known to have an unusual Q_B_ site in that it is relatively resistant to substitution of quinone with DCMU (Treves et al., 2016), up to 10 mM in our experience. The differential PSII efficiency between these two species in the addition of DCBQ alludes to appreciable differences in their acceptor side structures. These differences would feed into the largest single component of modulation in PSII-CEF: the redox poise of the PQ pool. We see in **Figure 3a** rise in F_v_/F_m_ over the course of flash trains in either growth light condition. This difference is likely an effect of state transitions, where the phosphorylation of LHCII by a mobile kinase works to match the rates between the two photosystems by detaching LHC proteins from one photosystem so that they may associate with the other (Wollman, 2001; Allen, 2003; Murata, 2009; Simkin et al., 2022). In low light or shaded conditions, the distribution of LHCs is in state 1 (associated with PSII), and in high intensity or high energy light, there is a shift to state 2 (LHCs associated with PSI). The removal of antenna over the course of the flash trains, long enough for this to occur in *C. ohadii* (Ananyev et al., 2017), would effectively lower any carotenoid-based quenching (qE) and diminish excitons reaching PSII. Conversely, the shift in the sigmoidal shape between light conditions suggests a significant change in the available PSII: PQ ratio (Ksas et al., 2022). It seems clear by the significant differences in FRR, PsbA concentration, oxygen evolution, and cyt *b_6_f* in the EL grown cultures that the PSII supercomplex components are differentially expressed, with a conserved property of exceptional photoprotection that is better modulated by *C. ohadii* specifically. Detailing the physiology and protein environment of PSII in *Chlorella ohadii* will be integral to our understanding of the unique nature of this unprecedented photoprotection.

When subjected to extreme light and high availability of CO_2_, *C. sorokiniana* replicates on timescales (minimum average doubling time of 1.8 h) that now places it amongst the fastest growing phototrophs on record, only exceeded by *C. ohadii* and the cyanobacterium *Synechococcus elongatus* UTEX 2973 (Ananyev et al., 2017; Ungerer et al., 2018). It should be noted that while *C. sorokiniana* has previously been grown in high light conditions (≥1500 µmol photons m^-2^ s^-1^) (Kliphuis et al., 2011; Holdmann et al., 2018), a comprehensive study of the PETC at this light intensity has not yet been reported as a direct comparison to related green algae. The capability of exceptional growth, originally thought to be a unique attribute of *C. ohadii*, seems to be more broadly applicable to the relatives of *Chlorella* and potentially *Picochlorum* (Foflonker et al., 2016; Gates et al., 2024; Krishnan et al., 2025), while the level of photochemistry by PSII in *C. ohadii* remains unmatched. Historically, the *Chlorella* genus is recognized for its exceptional carbon concentrating mechanism (CCM), which alleviates the rate limitation of the dark reactions of photosynthesis by concentrating carbon dioxide around RuBisCO (Raven et al., 2008; Ashour et al., 2024). This process is especially elevated in environments where CO_2_ availability is limited, such as the biological soil crust from which *Chlorella ohadii* was isolated. Assuming a conserved CCM between these *Chlorella* species, their differences in growth should arise due to their individual PETC efficiencies. When comparing the PETC of the EL *Chlorella*, the primary advantage of *C. ohadii* over *C. sorokiniana* likely lies in a differing molecular architecture surrounding P680, allowing for a significantly higher occurrence of successful charge separation in PSII. This distinction in more successful PSII operation by *C. ohadii* is directly illustrated in the requirement of DCBQ for oscillatory behavior in the FRR of EL *C. sorokiniana*. The only two existing structures of *C. ohadii* PSII (PDB: 8BD3) (Fadeeva et al., 2023) and (PDB: 9HD7) (Arshad et al., 2026) show multiple photoprotective proteins surrounding the WOC, but there are not currently available structures of related *Chlorella* for comparison, and both of these existing structures were extracted from cultures grown in low light conditions. Obtaining atomic structures of extreme-light acclimated *Chlorella* proteins for comparison in the future would be integral to our fundamental understanding of these processes.

## 5 Conclusions

At LL, the two closely related *Chlorella* possess similar WOC efficiency, LEF, redox potential of the PQ pool, amplitude of charge separation, ΔpH, rate oximetry, and amplitude of PSI usage. The PETC of *C. ohadii and C. sorokiniana* grown at LL are so congruent that they are not statistically distinguishable from each other. However, when grown in EL conditions, there are conserved changes to the redox potential of the PQ pool including a majority of reaction centers actively performing primary electron transfer and slow secondary electron transfer kinetics. *C. ohadii* best matches its trans-thylakoid membrane potential (via a similar amplitude of ECS) before and after the initiation of carbon fixation, while *C. sorokiniana* comparative has a significantly larger pmf at equilibrium and a delayed onset of carbon fixation. Overall, while it appears that *Chlorella ohadii* has an intrinsically more fine-tuned PSII structure to perform PSII-CEF and protect the integrity of the PETC compared to the acclimated *C. sorokiniana*, at least part of the *Chlorella* clade that includes these species has an underlying basis of photoprotection that can be acclimated. One important conserved kinetic in the investigated species is the average rate between charge separations in the EL grown cultures. Each of these rates, around 13 to 14 ms, are faster than the average turnover of the PQ pool, requiring a modification to the turnover of the pool to achieve faster charge transfer from P680. This is strong evidence of PSII-CEF being amplified in these organisms in EL conditions, although quantification of PSII-CEF is currently a technical challenge that will continue to be addressed in aims to validate the holistic findings we have observed herein. What can be more readily investigated and supported through external works is that PSI-CEF is also employed in both of these species, and strongly operating in *C. sorokiniana*. This differential approach to extreme photoprotection might be advantageous for incorporating fast growth to other green algae of biotechnical interest. These adapted features may also eventually prove imperative to understanding how light and drought tolerance can be transferred to the phototrophs that will continue to sustain the primary production of chemical energy we rely on in an ever-changing global environment.

## Data Availability

The raw data supporting the conclusions of this article will be made available by the authors, without undue reservation.

## References

[B1] Allen J. F. (2003). State transitions--a question of balance. Science 299, 1530–1532. doi: 10.1126/science.1082833 12624254

[B2] Ananyev G. Dismukes G. C. (2005). How fast can photosystem II split water? Kinetic performance at high and low frequencies. Photosynth. Res. 84, 355–365. doi: 10.1007/s11120-004-7081-1 16049797

[B3] Ananyev G. Gates C. Dismukes G. C. (2016). The oxygen quantum yield in diverse algae and cyanobacteria is controlled by partitioning of flux between linear and cyclic electron flow within photosystem II. Biochim. Biophys. Acta (BBA) - Bioenergetics 1857, 1380–1391. doi: 10.1016/j.bbabio.2016.04.056 27117512

[B4] Ananyev G. Gates C. Kaplan A. Dismukes G. C. (2017). Photosystem II-cyclic electron flow powers exceptional photoprotection and record growth in the microalga Chlorella ohadii. Biochim. Biophys. Acta (BBA) - Bioenergetics 1858, 873–883. doi: 10.1016/j.bbabio.2017.07.001 28734933

[B5] Ananyev G. Veldhuis M.-C. Zournas A. Gates C. Dismukes G. C. (2026). An optimized fast repetition rate fluorometry method reveals photochemical flux bottlenecks in C3 photosynthesis that limit carboxylation and growth. Mol. Plant. doi: 10.1016/j.molp.2026.05.023 42231573

[B6] Araki N. Takeda H. (1992). Species-specificity of the lytic activity of the cell wall in the genus Chlorella. Physiol. Plant 85, 704–709. doi: 10.1111/j.1399-3054.1992.tb04774.x 40046247

[B7] Aro E.-M. Virgin I. Andersson B. (1993). Photoinhibition of photosystem II. Inactivation, protein damage and turnover. Biochim. Biophys. Acta (BBA) - Bioenergetics 1143, 113–134. doi: 10.1016/0005-2728(93)90134-2 8318516

[B8] Arshad R. Skalidis I. Kopečný D. Brabencová S. Opatíková M. Ilík P. . (2026). Cryo-EM structure of photosystem II supercomplex from a green microalga with extreme phototolerance. Nat. Commun. 17, 341. doi: 10.1038/s41467-025-65861-2 41513636 PMC12789144

[B9] Ashour M. Mansour A. T. Alkhamis Y. A. Elshobary M. (2024). Usage of Chlorella and diverse microalgae for CO2 capture - towards a bioenergy revolution. Front. Bioeng. Biotechnol. 12, 2024. doi: 10.3389/fbioe.2024.1387519 39229458 PMC11368733

[B10] Azaman S. N. A. Nagao N. Yusoff F. M. Tan S. W. Yeap S. K. (2017). A comparison of the morphological and biochemical characteristics of Chlorella sorokiniana and Chlorella zofingiensis cultured under photoautotrophic and mixotrophic conditions. PeerJ 5, e3473. doi: 10.7717/peerj.3473 28929006 PMC5592954

[B11] Barber J. (2003). Photosystem II: the engine of life. Q. Rev. Biophys. 36, 71–89. doi: 10.1017/S0033583502003839 12643043

[B12] Bassham J. A. Benson A. A. Kay L. D. Harris A. Z. Wilson A. T. Calvin M. (1954). The path of carbon in photosynthesis. XXI. The cyclic regeneration of carbon dioxide acceptor1. J. Am. Chem. Soc 76, 1760–1770. doi: 10.1021/ja01636a012

[B13] Baudelet P.-H. Ricochon G. Linder M. Muniglia L. (2017). A new insight into cell walls of Chlorophyta. Algal Res. 25, 333–371. doi: 10.1016/j.algal.2017.04.008 38826717

[B14] Bhattacharjee S. Neese F. Pantazis D. A. (2023). Triplet states in the reaction center of photosystem II. Chem. Sci. 14, 9503–9516. doi: 10.1039/D3SC02985A 37712047 PMC10498673

[B15] Blomberg M. R. A. Siegbahn P. E. M. Babcock G. T. (1998). Modeling electron transfer in biochemistry: a quantum chemical study of charge separation in Rhodobacter sphaeroides and photosystem II. J. Am. Chem. Soc 120, 8812–8824. doi: 10.1021/ja9805268

[B16] Borowitzka M. A. (2013). High-value products from microalgae—their development and commercialisation. J. Appl. Phycol. 25, 743–756. doi: 10.1007/s10811-013-9983-9 30311153

[B17] Bouchard J. N. Roy S. Campbell D. A. (2006). UVB effects on the photosystem II-D1 protein of phytoplankton and natural phytoplankton communities. Photochem. Photobiol. 82, 936–951. doi: 10.1562/2005-08-31-ir-666 16620154

[B18] Bradford M. M. (1976). A rapid and sensitive method for the quantitation of microgram quantities of protein utilizing the principle of protein-dye binding. Anal. Biochem. 72, 248–254. doi: 10.1016/0003-2697(76)90527-3 942051

[B19] Broess K. Trinkunas G. van der Weij-de Wit C. D. Dekker J. P. van Hoek A. van Amerongen H. (2006). Excitation energy transfer and charge separation in photosystem II membranes revisited. Biophys. J. 91, 3776–3786. doi: 10.1529/biophysj.106.085068 16861268 PMC1630486

[B20] Campbell D. A. Cockshutt A. M. Porankiewicz-Asplund J. (2003). Analysing photosynthetic complexes in uncharacterized species or mixed microalgal communities using global antibodies. Physiol. Plant 119, 322–327. doi: 10.1034/j.1399-3054.2003.00175.x 42325892

[B21] Cardona T. Sedoud A. Cox N. Rutherford A. W. (2012). Charge separation in photosystem II: a comparative and evolutionary overview. Biochim. Biophys. Acta (BBA) - Bioenergetics 1817, 26–43. doi: 10.1016/j.bbabio.2011.07.012 21835158

[B22] Chen C. Tang T. Shi Q. Zhou Z. Fan J. (2022). The potential and challenge of microalgae as promising future food sources. Trends Food Sci. Technol. 126, 99–112. doi: 10.1016/j.tifs.2022.06.016 38826717

[B23] Dekker J. P. Plijter J. J. Ouwehand L. Van Gorkom H. J. (1984). Kinetics of manganese redox transitions in the oxygen-evolving apparatus of photosynthesis. Biochim. Biophys. Acta (BBA)-Bioenergetics 767, 176–179. doi: 10.1016/0005-2728(84)90093-8

[B24] Dekker J. P. Van Grondelle R. (2000). Primary charge separation in photosystem II. Photosynth. Res. 63, 195–208. doi: 10.1023/A:1006468024245 16228430

[B25] Fadeeva M. Klaiman D. Caspy I. Nelson N. (2023). Structure of Chlorella ohadii photosystem II reveals protective mechanisms against environmental stress. Cells 12, 1971. doi: 10.3390/cells12151971 37566050 PMC10416949

[B26] Foflonker F. Ananyev G. Qiu H. Morrison A. Palenik B. Dismukes G. C. . (2016). The unexpected extremophile: tolerance to fluctuating salinity in the green alga Picochlorum. Algal Res. 16, 465–472. doi: 10.1016/j.algal.2016.04.003 38826717

[B27] Fu H.-Y. Picot D. Choquet Y. Longatte G. Sayegh A. Delacotte J. . (2017). Redesigning the QA binding site of photosystem II allows reduction of exogenous quinones. Nat. Commun. 8, 15274. doi: 10.1038/ncomms15274 28466860 PMC5418674

[B28] Garab G. (2025). Revisiting the QA model of chlorophyll-a fluorescence induction: new perspectives to monitor the photochemical activity and structural dynamics of photosystem II. Photosynth. Res. 163, 54. doi: 10.1007/s11120-025-01178-x 41128974 PMC12549761

[B29] Gates C. Ananyev G. Dismukes G. C. (2020). Realtime kinetics of the light driven steps of photosynthetic water oxidation in living organisms by “stroboscopic” fluorometry. Biochim. Biophys. Acta (BBA) - Bioenergetics 1861, 148212. doi: 10.1016/j.bbabio.2020.148212 32320684

[B30] Gates C. Ananyev G. Foflonker F. Bhattacharya D. Dismukes G. C. (2024). Exceptional quantum efficiency powers biomass production in halotolerant algae Picochlorum sp. Photosynth. Res. 162, 439–457. doi: 10.1007/s11120-024-01075-9 38329705

[B31] Gorbunov M. Y. Kolber Z. S. Falkowski P. G. (1999). Measuring photosynthetic parameters in individual algal cells by fast repetition rate fluorometry. Photosynth. Res. 62, 141–153. doi: 10.1023/a:1006360005033 41886696

[B32] Graan T. Ort D. R. (1986). Detection of oxygen-evolving photosystem II centers inactive in plastoquinone reduction. Biochim. Biophys. Acta (BBA)-Bioenergetics 852, 320–330. doi: 10.1016/0005-2728(86)90238-0

[B33] Holdmann C. Schmid-Staiger U. Hornstein H. Hirth T. (2018). Keeping the light energy constant — cultivation of Chlorella sorokiniana at different specific light availabilities and different photoperiods. Algal Res. 29, 61–70. doi: 10.1016/j.algal.2017.11.005 38826717

[B34] Jugulam M. Pandian B. A. Prasad P. V. V. (2025). Overview of metabolism-based resistance to PS - II inhibitors in weed species. Resistance Weeds Herbicide Metab., 217–232. doi: 10.1002/9781119686699.ch11 41531421

[B35] Kamada S. Nakajima Y. Shen J.-R. (2023). Structural insights into the action mechanisms of artificial electron acceptors in photosystem II. J. Biol. Chem. 299. doi: 10.1016/j.jbc.2023.104839 37209822 PMC10300377

[B36] Kessler E. Huss V. A. R. (1992). Comparative physiology and biochemistry and taxonomic assignment of the Chlorella (Chlorophyceae) strains of the culture collection of the University of Texas at Austin. J. Phycol. 28, 550–553. doi: 10.1111/j.0022-3646.1992.00550.x 40046247

[B37] Kliphuis A. M. J. Janssen M. van den End E. J. Martens D. E. Wijffels R. H. (2011). Light respiration in Chlorella sorokiniana. J. Appl. Phycol. 23, 935–947. doi: 10.1007/s10811-010-9614-7 22131644 PMC3210360

[B38] Kramer D. M. Sacksteder C. A. (1998). A diffused-optics flash kinetic spectrophotometer (DOFS) for measurements of absorbance changes in intact plants in the steady-state. Photosynth. Res. 56, 103–106. doi: 10.1023/A:1005968211506 16228356

[B39] Krieger-Liszkay A. (2004). Singlet oxygen production in photosynthesis. J. Exp. Bot. 56, 337–346. doi: 10.1093/jxb/erh237 15310815

[B40] Krishnan A. Dahlin L. R. Guarnieri M. T. Weissman J. C. Posewitz M. C. (2025). Small cells with big photosynthetic productivities: biotechnological potential of the Picochlorum genus. Trends Biotechnol. 43, 759–772. doi: 10.1016/j.tibtech.2024.10.004 39521625

[B41] Ksas B. Alric J. Caffarri S. Havaux M. (2022). Plastoquinone homeostasis in plant acclimation to light intensity. Photosynth. Res. 152, 43–54. doi: 10.1007/s11120-021-00889-1 35000138

[B42] Kumar K. Dasgupta C. N. Das D. (2014). Cell growth kinetics of Chlorella sorokiniana and nutritional values of its biomass. Bioresour. Technol. 167, 358–366. doi: 10.1016/j.biortech.2014.05.118 24997380

[B43] Lamb J. J. Røkke G. Hohmann-Marriott M. F. (2018). Chlorophyll fluorescence emission spectroscopy of oxygenic organisms at 77 K. Photosynthetica 56, 105–124. doi: 10.1007/s11099-018-0791-y 30311153

[B44] Lizzul A. M. Lekuona-Amundarain A. Purton S. Campos L. C. (2018). Characterization of chlorella sorokiniana, UTEX 1230. Biology 7, 25. doi: 10.3390/biology7020025 29652809 PMC6023026

[B45] MacKenzie T. D. Johnson J. M. Cockshutt A. M. Burns R. A. Campbell D. A. (2005). Large reallocations of carbon, nitrogen, and photosynthetic reductant among phycobilisomes, photosystems, and Rubisco during light acclimation in Synechococcus elongatus strain PCC7942 are constrained in cells under low environmental inorganic carbon. Arch. Microbiol. 183, 190–202. doi: 10.1007/s00203-005-0761-1 15726330

[B46] Milledge J. J. (2011). Commercial application of microalgae other than as biofuels: a brief review. Rev. Environ. Sci. Bio/Technol. 10, 31–41. doi: 10.1007/s11157-010-9214-7 30311153

[B47] Mino H. Kawamori A. (2001). EPR studies of the water oxidizing complex in the S1 and the higher S states: the manganese cluster and YZ radical. Biochim. Biophys. Acta (BBA) - Bioenergetics 1503, 112–122. doi: 10.1016/S0005-2728(00)00229-2 11115628

[B48] Morita M. Watanabe Y. Saiki H. (2000). High photosynthetic productivity of green microalga Chlorella sorokiniana. Appl. Biochem. Biotechnol. 87, 203–218. doi: 10.1385/ABAB:87:3:203 10982230

[B49] Murata N. (2009). The discovery of state transitions in photosynthesis 40 years ago. Photosynth. Res. 99, 155–160. doi: 10.1007/s11120-008-9389-8 19037742

[B50] Murik O. Geffen O. Shotland Y. Fernandez-Pozo N. Ullrich K. K. Walther D. . (2024). Genomic imprints of unparalleled growth. New Phytol. 241, 1144–1160. doi: 10.1111/nph.19444 38072860

[B51] Oliver T. Kim T. D. Trinugroho J. P. Cordón-Preciado V. Wijayatilake N. Bhatia A. . (2023). The evolution and evolvability of photosystem II. Annu. Rev. Plant Biol. 74, 225–257. doi: 10.1146/annurev-arplant-070522-062509 36889003

[B52] Oncel S. S. (2013). Microalgae for a macroenergy world. Renewable Sustain. Energy Rev. 26, 241–264. doi: 10.1016/j.rser.2013.05.059 38826717

[B53] Porra R. Thompson W. (1989). Determination of accurate extinction coefficients and simultaneous equations for assaying chlorophylls a and b extracted with four different solvents: verification of the …. Biochim. Biophys. Acta. 975 (3), 384–394. doi: 10.1016/S0005-2728(89)80347-0

[B54] Pospíšil P. (2012). Molecular mechanisms of production and scavenging of reactive oxygen species by photosystem II. Biochim. Biophys. Acta (BBA) - Bioenergetics 1817, 218–231. doi: 10.1016/j.bbabio.2011.05.017 21641332

[B55] Raven J. A. Cockell C. S. De La Rocha C. L. (2008). The evolution of inorganic carbon concentrating mechanisms in photosynthesis. Philos. Trans. R. Soc. B. Biol. Sci. 363, 2641–2650. doi: 10.1098/rstb.2008.0020 18487130 PMC2606764

[B56] Renger T. Schlodder E. (2011). Optical properties, excitation energy and primary charge transfer in photosystem II: Theory meets experiment. J. Photochem. Photobiol. B. Biol. 104, 126–141. doi: 10.1016/j.jphotobiol.2011.03.016 21531572

[B57] Rippka R. Deruelles J. Waterbury J. B. Herdman M. Stanier R. Y. (1979). Generic assignments, strain histories and properties of pure cultures of cyanobacteria. Microbiology 111, 1–61. doi: 10.1099/00221287-111-1-1 27077644

[B58] Santabarbara S. Bordignon E. Jennings R. C. Carbonera D. (2002). Chlorophyll triplet states associated with photosystem II of thylakoids. Biochemistry 41, 8184–8194. doi: 10.1021/bi0201163 12069611

[B59] Shen J.-R. (2015). The structure of photosystem II and the mechanism of water oxidation in photosynthesis. Annu. Rev. Plant Biol. 66, 23–48. doi: 10.1146/annurev-arplant-050312-120129 25746448

[B60] Simkin A. J. Kapoor L. Doss C. G. P. Hofmann T. A. Lawson T. Ramamoorthy S. (2022). The role of photosynthesis related pigments in light harvesting, photoprotection and enhancement of photosynthetic yield in planta. Photosynth. Res. 152, 23–42. doi: 10.1007/s11120-021-00892-6 35064531

[B61] Spolaore P. Joannis-Cassan C. Duran E. Isambert A. (2006). Commercial applications of microalgae. J. Biosci. Bioeng. 101, 87–96. doi: 10.1263/jbb.101.87 16569602

[B62] Steiner G. Saini D. Gates C. (2026). Extreme light acclimation reveals inherent photoprotective mechanisms in a natively low-light Chlorella. Algal Res. 96, 104727. doi: 10.1016/j.algal.2026.104727 38826717

[B63] Steiner G. Saini D. Kapoor A. Gates C. (2025). Adaptation of the Chlorella photosynthetic electron transport chain to environmental light conditions. Algal Res. 91, 104223. doi: 10.1016/j.algal.2025.104223 38826717

[B64] Takeuchi K. Harimoto S. Maekawa S. Miyake C. Ifuku K. (2025). PSII photoinhibition as a protective strategy: maintaining an oxidative state of PSI by suppressing PSII activity under environmental stress. Physiol. Plant 177, e70392. doi: 10.1111/ppl.70392 40635124

[B65] Thoré E. S. J. Muylaert K. Bertram M. G. Brodin T. (2023). Microalgae. Curr. Biol. 33, R91–R95. doi: 10.1016/j.cub.2022.12.032 36750029

[B66] Treves H. Raanan H. Finkel O. M. Berkowicz S. M. Keren N. Shotland Y. . (2013). A newly isolated Chlorella sp. from desert sand crusts exhibits a unique resistance to excess light intensity. FEMS Microbiol. Ecol. 86, 373–380. doi: 10.1111/1574-6941.12162 23773145

[B67] Treves H. Raanan H. Kedem I. Murik O. Keren N. Zer H. . (2016). The mechanisms whereby the green alga Chlorella ohadii, isolated from desert soil crust, exhibits unparalleled photodamage resistance. New Phytol. 210, 1229–1243. doi: 10.1111/nph.13870 26853530

[B68] Ungerer J. Lin P.-C. Chen H.-Y. Pakrasi H. B. (2018). Adjustments to photosystem stoichiometry and electron transfer proteins are key to the remarkably fast growth of the cyanobacterium Synechococcus elongatus UTEX 2973. mBio 9, 10.1128/mbio.02327–02317. doi: 10.1128/mbio.02327-17 29437923 PMC5801466

[B69] Vass I. (2012). Molecular mechanisms of photodamage in the photosystem II complex. Biochim. Biophys. Acta (BBA)-Bioenergetics 1817, 209–217. doi: 10.1016/j.bbabio.2011.04.014 21565163

[B70] Vinyard D. J. Ananyev G. M. Charles Dismukes G. (2013a). Photosystem II: the reaction center of oxygenic photosynthesis. Annu. Rev. Biochem. 82, 577–606. doi: 10.1146/annurev-biochem-070511-100425 23527694

[B71] Vinyard D. J. Zachary C. E. Ananyev G. Dismukes G. C. (2013b). Thermodynamically accurate modeling of the catalytic cycle of photosynthetic oxygen evolution: a mathematical solution to asymmetric Markov chains. Biochim. Biophys. Acta 1827, 861–868. doi: 10.1016/j.bbabio.2013.04.008 23643726

[B72] Wang N. Pei H. Xiang W. Li T. Lin S. Wu J. . (2023). Rapid screening of microalgae as potential sources of natural antioxidants. Foods 12, 2652. doi: 10.3390/foods12142652 37509744 PMC10378671

[B73] Wasielewski M. R. Johnson D. G. Govindjee Preston C. Seibert M. (1989). Determination of the primary charge separation rate in photosystem II reaction centers at 15 K. Photosynth. Res. 22, 89–99. doi: 10.1007/BF00114769 24424681

[B74] Wollman F. A. (2001). State transitions reveal the dynamics and flexibility of the photosynthetic apparatus. EMBO J. 20, 3623–3630. doi: 10.1093/emboj/20.14.3623 11447103 PMC125556

[B75] Yun H.-S. Kim Y.-S. Yoon H.-S. (2020). Characterization of Chlorella sorokiniana and Chlorella vulgaris fatty acid components under a wide range of light intensity and growth temperature for their use as biological resources. Heliyon 6. doi: 10.1016/j.heliyon.2020.e04447 32743091 PMC7387821

