# Supplementary material for: Distinctions in photochemistry between Chlorella ohadii and Chlorella sorokiniana are dependent on growth light intensity
Source: Front Plant Sci. 2026 Jul 29;17:1858699. doi: 10.3389/fpls.2026.1858699 (PMC13461607; doi:10.3389/fpls.2026.1858699)
Supplement: Supplementary file 1 [file DataSheet1.pdf]

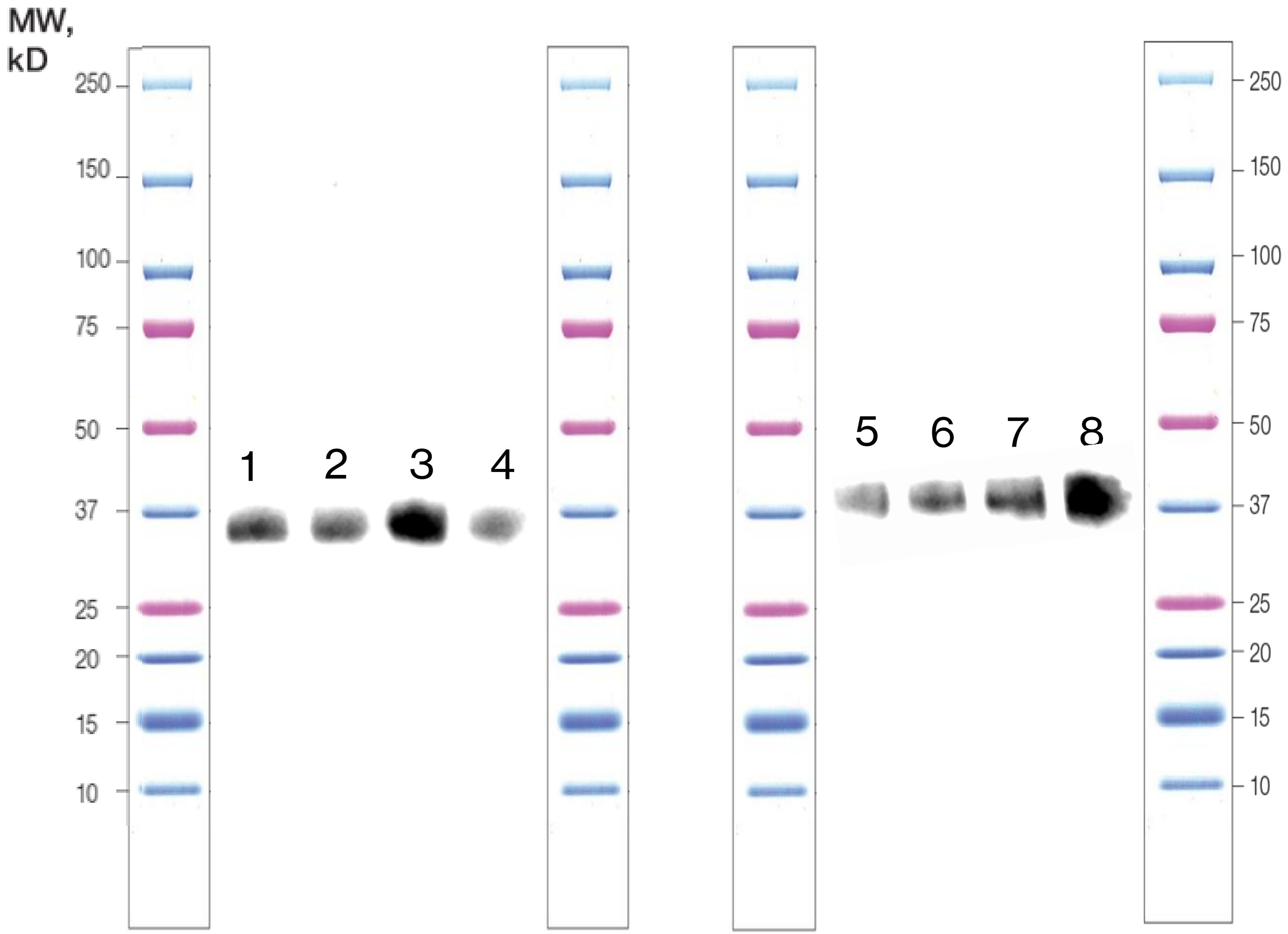

Supplementary Figure S1. **Western Blotting of D1 in *Chlorella ohadii* and *Chlorella sorokiniana*.** Western blot images of PsbA (D1) in each related *Chlorella* species and growth light condition. Sample lanes were loaded with 1.2 µg of isolated protein extract from each culture. Lanes 1-4 - Samples: 1- EL-grown *Chlorella ohadii* (2,000 µmol photons m^{-2} s^{-1}); 2- LL-grown *Chlorella ohadii* (20 µmol photons m^{-2} s^{-1}); 3- EL-grown *C. sorokiniana*4- LL-grown *C. sorokiniana*; Lanes 5-8 - PsbA standards (pmol): 5- 0.050; 6- 0.150; 7- 0.300; 8- 0.450.

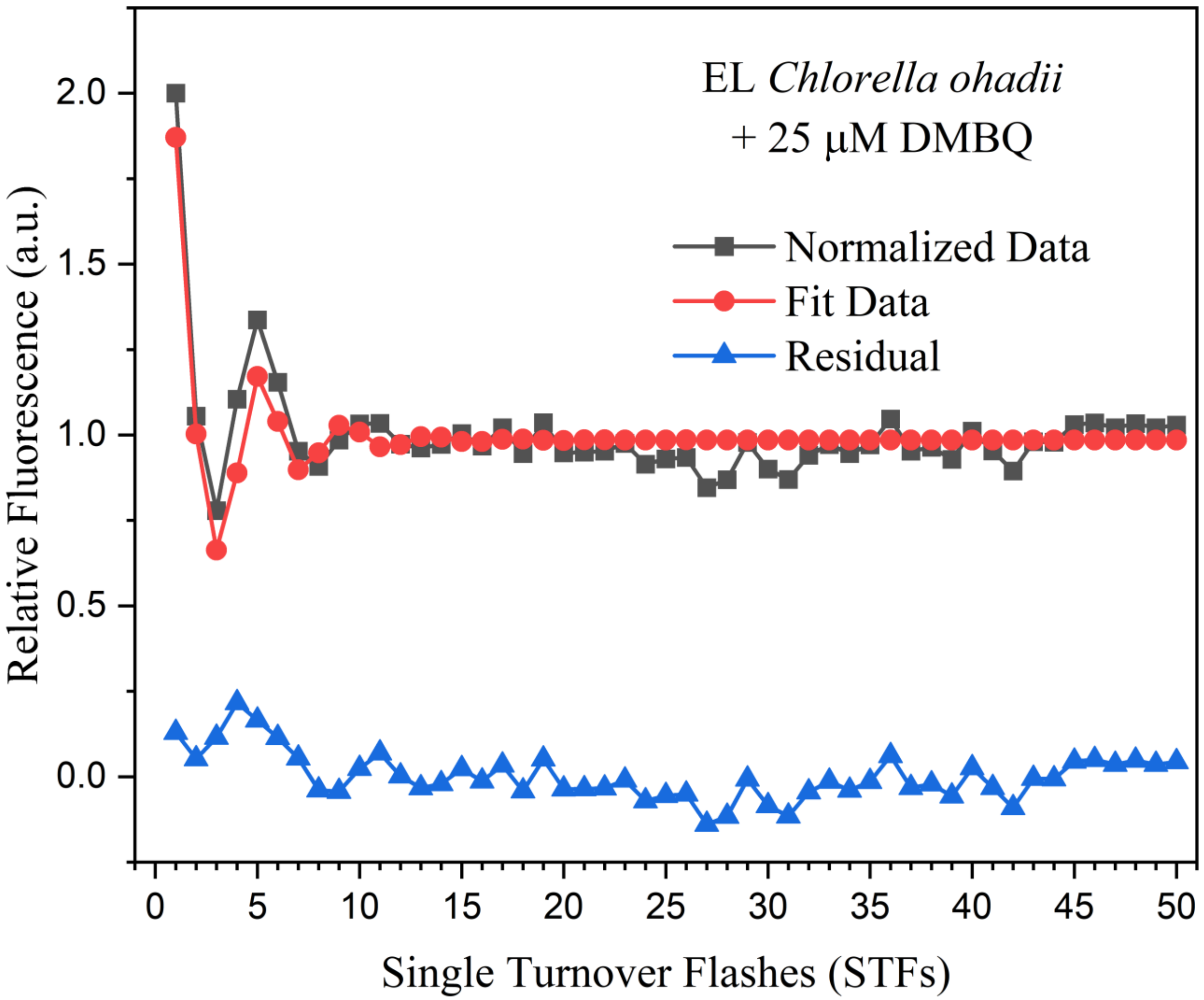

Supplementary Figure S2. **Fast Repetition Rate Fluorometry of *Chlorella ohadii* with Exogenous Quinone.** Raw 10 Hz fast repetition rate fluorometry values are normalized (black trace), fit (red trace), and residual (blue trace) of extreme light grown *Chlorella ohadii* with 25 µM DMBQ added.

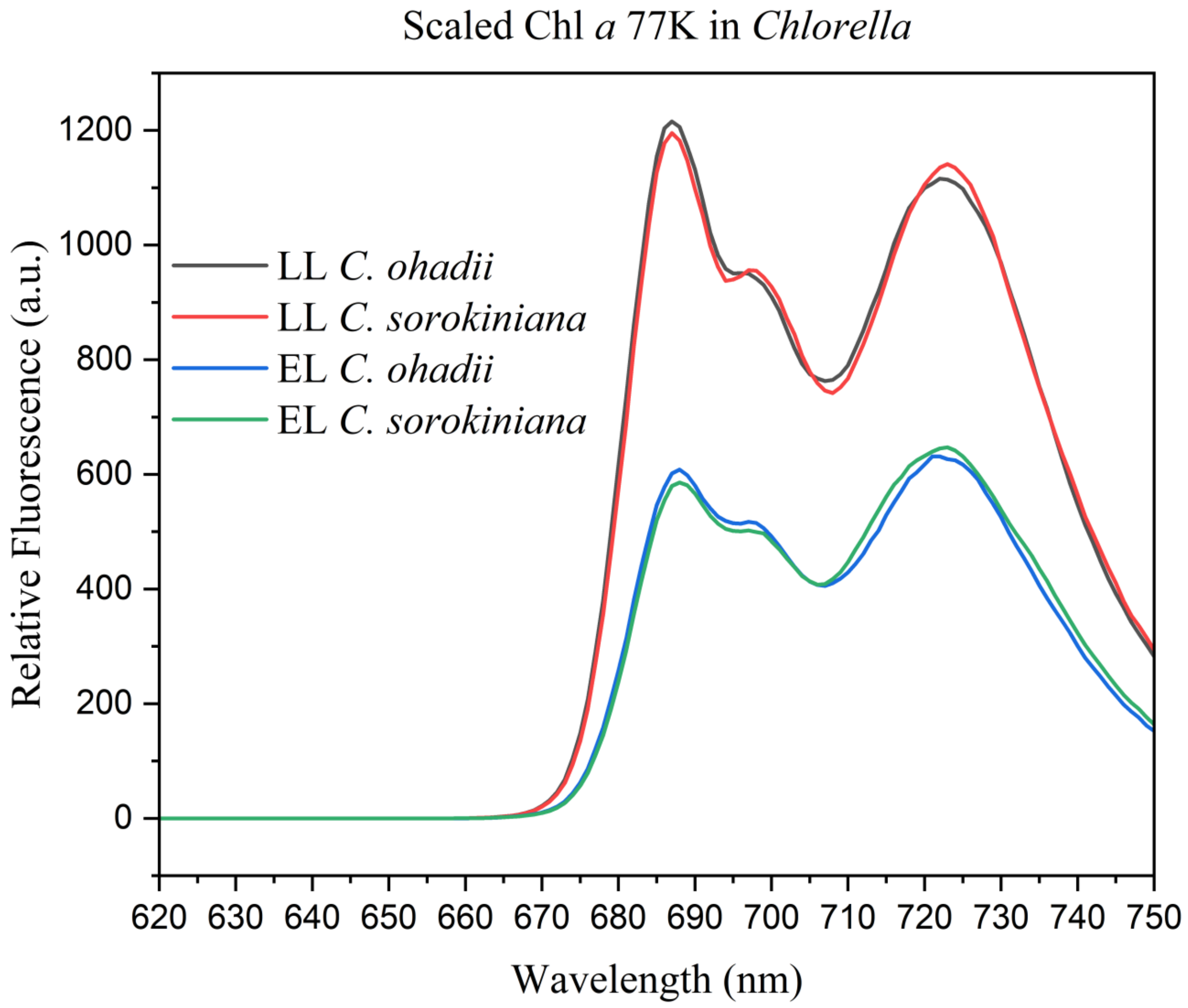

Supplementary Figure S3. **Scaled Cryogenic Fluorescence in *Chlorella* Cells.** Modification of **Fig. 4A**, where the spectra of *Chlorella ohadii* (black and blue traces) have each been scaled approximately 5% away from the midline of the y-axis, showcasing that the relative distribution of photosystems in each *Chlorella* is nearly equivalent between species at either growth light condition.
